# Surveillance of Noncommunicable Disease Epidemic Through the Integrated Noncommunicable Disease Collaborative Management System: Feasibility Pilot Study Conducted in the City of Ningbo, China

**DOI:** 10.2196/17340

**Published:** 2020-07-23

**Authors:** Sixuan Li, Liang Zhang, Shiwei Liu, Richard Hubbard, Hui Li

**Affiliations:** 1 Division of Chronic and Noncommunicable Disease Control and Prevention Ningbo Municipal Center for Disease Control and Prevention Ningbo China; 2 Division of Big Data Ningbo Municipal Center for Disease Control and Prevention Ningbo China; 3 National Center for Chronic and Noncommunicable Disease Control and Prevention Chinese Center for Disease Control and Prevention Beijing China; 4 Division of Epidemiology and Public Health University of Nottingham Nottingham United Kingdom

**Keywords:** surveillance, noncommunicable diseases, regional health information platform, electronic health records.

## Abstract

**Background:**

Noncommunicable diseases (NCDs) have become the main public health concern worldwide. With rapid economic development and changes in lifestyles, the burden of NCDs in China is increasing dramatically every year. Monitoring is a critical measure for NCDs control and prevention. However, because of the lack of regional representativeness, unsatisfactory data quality, and inefficient data sharing and utilization, the existing surveillance systems and surveys in China cannot track the status and transition of NCDs epidemic.

**Objective:**

To efficaciously track NCDs epidemic in China, this pilot program conducted in Ningbo city by the Chinese Center for Disease Control and Prevention (CDC) aimed to develop an innovative model for NCDs surveillance and management: the integrated noncommunicable disease collaborative management system (NCDCMS).

**Methods:**

This Ningbo model was designed and developed through a 3-level (county/district, municipal, and provincial levels) direct reporting system based on the regional health information platform. The uniform data standards and interface specifications were established to connect different platforms and conduct data exchanges. The performance of the system was evaluated based on the 9 attributes of surveillance system evaluation framework recommended by the US CDC.

**Results:**

NCDCMS allows automatic NCDs data exchanging and sharing via a 3-level public health data exchange platform in China. It currently covers 201 medical institutions throughout the city. Compared with previous systems, automatic popping up of the report card, automatic patient information extraction, and real-time data exchange process have highly improved the simplicity and timeliness of the system. The data quality meets the requirements to monitor the incidence trend of NCDs accurately, and the comprehensive data types obtained from the database (ie, directly from the 3-level platform on the data warehouse) also provide a useful information to conduct scientific studies. So far, 98.1% (201/205) of medical institutions across Ningbo having been involved in data exchanges with the model. Evaluations of the system performance showed that NCDCMS has high levels of simplicity, data quality, acceptability, representativeness, and timeliness.

**Conclusions:**

NCDCMS completely reshaped the process of NCD surveillance reporting and had unique advantages, which include reducing the work burden of different stakeholders by data sharing and exchange, eliminating unnecessary redundancies, reducing the amount of underreporting, and structuring population-based cohorts. The Ningbo model will be gradually promoted elsewhere following this success of the pilot project, and is expected to be a milestone in NCDs surveillance, control, and prevention in China.

## Introduction

With the rapid socioeconomic development and acceleration of population aging and urbanization, noncommunicable diseases (NCDs), such as cardiovascular diseases, cancers, chronic obstructive pulmonary diseases (COPDs), and diabetes, have become the main public health concerns worldwide. The WHO reported that NCDs kill around 40 million people every year, equivalent to 70% of all deaths globally [1]. China, as a developing country with the largest population, has been experiencing incremental prevalence of NCDs over the past decades; the proportional burden of NCDs increased by 37.27% from 60.21% in 1990 to 82.65% in 2016 [2]. Studies have shown that the prevalence of diabetes in adults was 9.7% in 2013, COPD among people aged 40 and above was 13.6% in 2015, and hypertension in adults aged 35-75 years was 44.7% in 2014-2017 in China [3-5]. Moreover, cardiovascular diseases, cancer, and chronic respiratory disease were the leading causes of death, accounting for approximately 79.4% of total deaths [6].

Monitoring is a critical and indispensable measure for NCDs control and prevention, including providing evidence for policy making and data support for evaluation of the global and regional targets [7]. As a cornerstone of public health practice in improving disease surveillance, the US Centers for Disease Control and Prevention (CDC) launched the CDC Surveillance Strategy in 2014 to achieve the targets of advancing the use of electronic health records (EHRs), retiring redundant systems, and maximizing performance of agency resources [8]. In China, the Chronic Disease and Risk Factor Surveillance Survey was introduced in 2004 using a multistage stratified cluster random sampling method to monitor NCD epidemics nationwide every 3 years [5,9-11]. However, due to the collection of NCD information by self-reports and the heavy workload involved in field survey, the monitoring data can only be obtained through selected surveillance points and their quality varies among regions. Several surveys and registries on specific chronic diseases are available in China, including those on stroke, hypertension, COPD, diabetes, and cancer, which provide valuable national epidemiological data on NCDs [3-5,12,13]. However, because these surveys and registries are independent from each other with inconsistent research methods and different population coverage, it is difficult to compare the varied results and obtain a whole picture of current situation of NCDs in China, which also reflects the shortcomings of traditional surveillance system in data sharing and utilization [14]. A population-based data with high quality generated from a reliable surveillance system is crucial to detect the priorities to track and tackle NCDs and find the cost-effective interventions to reduce the burden of NCDs.

Ningbo is a prefecture-level city of Zhejiang province in eastern China, consists of 10 districts with a registered population of 5.93 million, and has the very advanced regional health information platform in China. In 2016, Ningbo successfully achieved the 3-level (county/district, municipal, and provincial level) direct reporting of infectious diseases through a data exchange platform, which shortened the reporting time to 2 minutes from uploading a report card in the hospital information system (HIS) to the national information system [15]. Two years later, a similar but improved reporting and management model for NCDs was successfully implemented, named integrated noncommunicable disease collaborative management system (NCDCMS). This pilot study was conducted in Ningbo by the Chinese CDC and aimed to develop an innovative model for NCDs surveillance and management in order to efficaciously tackle NCDs epidemic in China.

## Methods

### Ningbo Model

The Ningbo model was constructed through a 3-level direct reporting strategy based on the regional health information platform. The uniform data standards and interface specifications were developed to connect different platforms and conduct data exchanges. NCDs registration report and management standards were issued to clarify data quality evaluation indicators and data security management standards. 9 attributes of the surveillance system evaluation framework recommended by the US CDC were used for evaluating the performance of NCDCMS [16,17].

### Architecture of the NCDCMS

**Figure 1:**
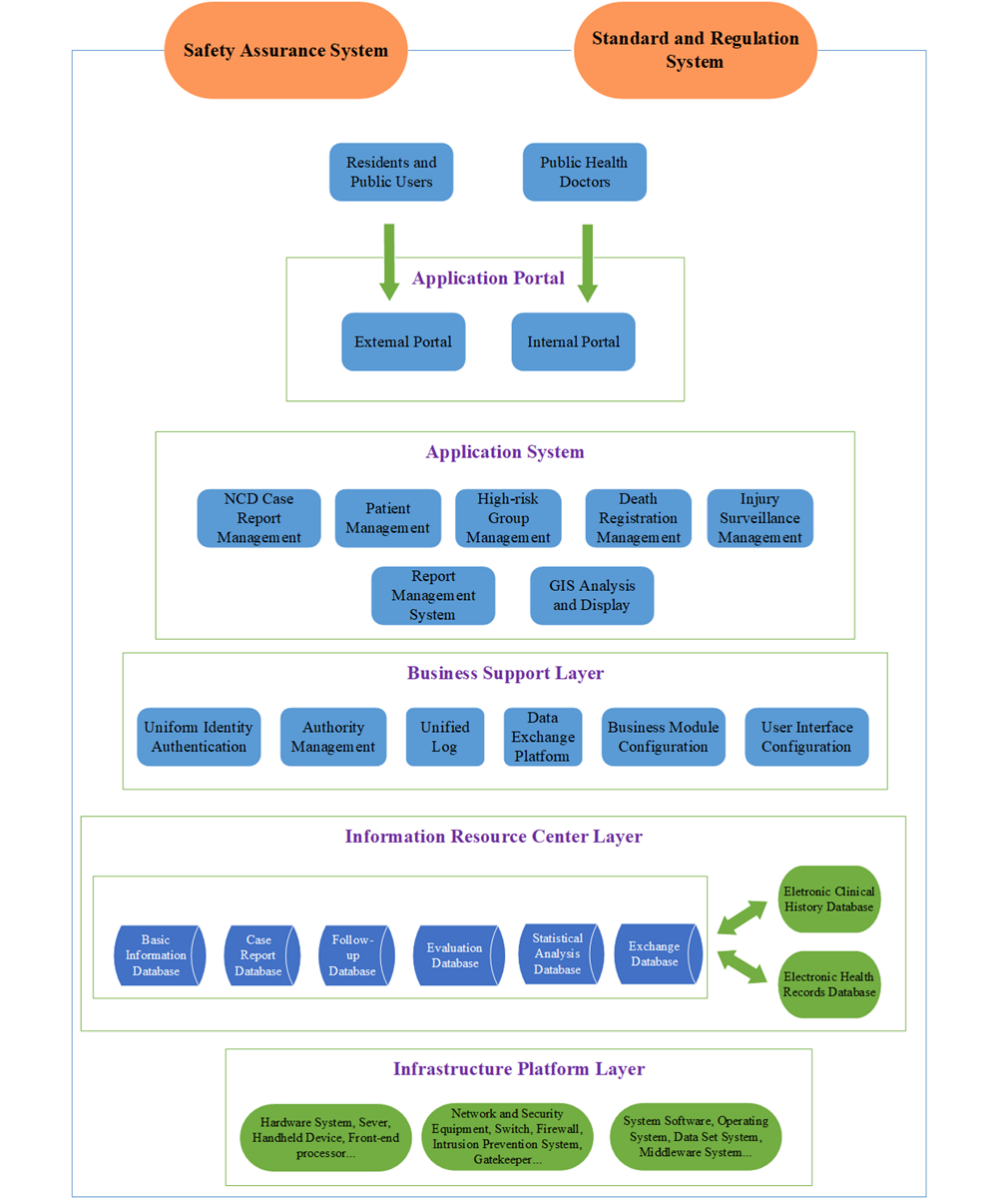
Architecture of the integrated noncommunicable disease collaborative management system. GIS: geographic information system;

With full consideration of the current situation of Ningbo and the potential requirements of system development, the SOA (service-oriented architecture) and ESB (enterprise service bus) technologies were used for building a multilayer architecture system based on the concept of integrated information system design. The architecture of NCDCMS is made up of 7 components: the infrastructure platform layer, the NCDs information resource center, the business support layer, the application system, the application portal, the safety assurance system, and the standard and regulation system. Figure 1 shows the architecture of the overall system.

### Process of Model Construction

To build an effective and reliable NCDs surveillance and management system, the NCDs 3-level direct reporting model was constructed by following these 4 aspects: (1) We completed the construction of the 3-level public health data exchange platform by connecting Ningbo Health Network (municipal level) with Zhejiang Provincial Health Network (province level) and County/District Health Network (county/district level). Health Network is the special information network for health industry in China. On this basis, the Ningbo public health data exchange platform was organized to achieve the data exchange from the county/district platform to the municipal platform, and further to the provincial platform. (2) We developed uniform data standards and interface specifications. The interface specifications were used as regulations and standards to connect different platforms and conduct data collection, exchange, inquiry, and reconciliation. (3) To connect HIS with the data exchange platform, all medical institutions were required to adjust their public health module in accordance with the uniform data standards and the interface specifications. After the adjustment, automatic popping up of report card, automatic extraction of patient information, and real-time data exchange among platforms can be achieved. (4) We finally issued the Ningbo NCDs Registration Report and Management Standards. It elaborated the standardized workflow and mechanisms for interdepartmental collaborations between different stakeholders. The indicators of data quality evaluation and data security management were also included and elaborated in detail in these standards.

### Evaluations of the System Performance

Because NCDCMS is a public health surveillance system, its performance should be evaluated to ensure the public health problems are being monitored efficiently and effectively. We evaluated the performance of the NCDCMS with the 9 surveillance system attributes based on the guidelines for evaluating public health surveillance systems developed by the US CDC: simplicity, flexibility, data quality, acceptability, sensitivity, predictive value positive, representativeness, timeliness, and stability [16]. As public health surveillance systems vary in objective, methods, purpose, and scope, attributes that are important in one system might be less important in another. Taking into account the purpose and characteristics of NCDCMS, we selected 6 attributes to evaluate the performance of NCDCMS. The definitions of these 6 attributes are presented in Table 1 [17].

**Table 1:** Definitions of surveillance system attributes.

Attributes	Definition
Simplicity	System’s structure and ease of operation
Flexibility	Ability to adapt to changing information needs or technological operating conditions with little additional time, personnel, or allocated funds
Data quality	Completeness and validity of the data recorded in the system
Acceptability	Willingness of persons and organizations to participate in the system
Representativeness	Ability to accurately describe the occurrence of a health-related event over time and its distribution in the population by place and person
Timeliness	Speed between steps in a system

### Ethical Considerations

Because the construction of NCDCMS is also a component of the Ningbo Smart City Project, it is a governmental initiative rather than a study. The ethical issues need to be addressed but could be different from general scientific studies. In the process of system construction, the protection of residents’ personal privacy has been fully taken into account. Moreover, the regional health information platform of Ningbo is nationally certified and has reached the maturity level of IV, which is considered the highest level of regional information protection. Thus, it is legal and safe to collect residents’ data through the system.

### Ethics Approval

The study was approved by the Ethics Review Committee of Ningbo Municipal Centers for Disease Control and Prevention.

### Availability of Data and Material

The data sets generated/analyzed during this study are not publicly available due to the protection of residents’ personal privacy but are available from the corresponding author on reasonable request.

## Results

### Data Collection and Exchange

In NCDCMS, 4 types of notifiable NCDs (diabetes mellitus, ischemic heart disease and cardiac arrest, cerebrovascular disease, malignant neoplasms, and benign neoplasm of central nervous system) report cards are collected through a stepwise 3-level public health data exchange platform. The types of NCDs and the corresponding ICD-10 codes are listed in Table 2. Besides, a vital registration system is integrated into NCDCMS, which is able to collect cause-of-death data accurately and efficiently, from which we can obtain insights into the underlying causes of death.

**Figure 2:**
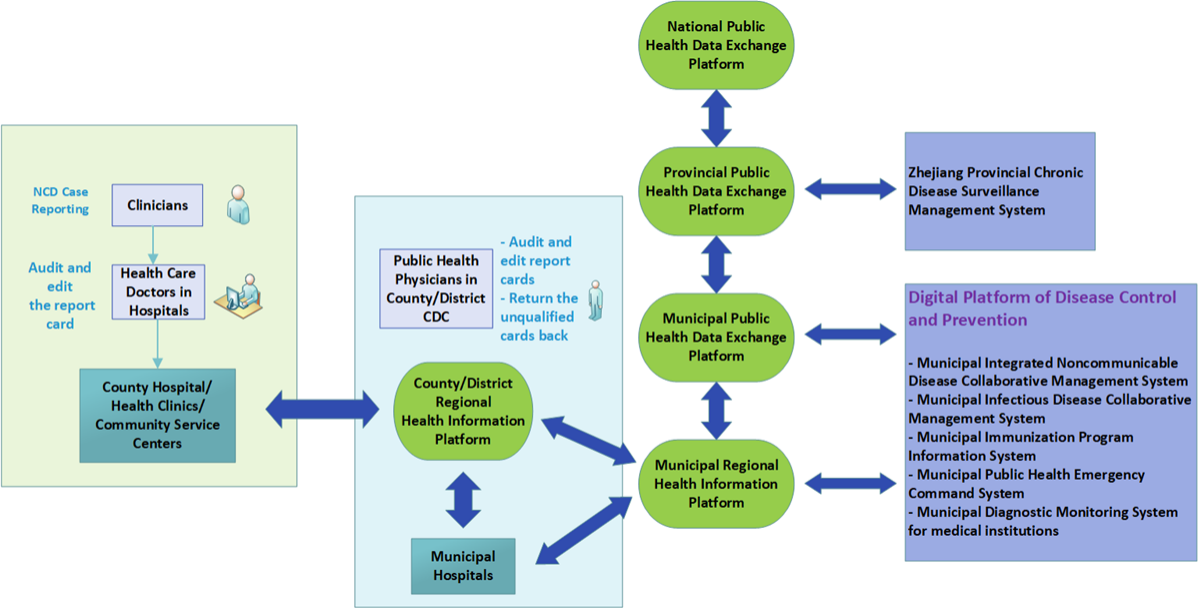
The process of data exchange and data sharing through a 3-level (county, district, and municipal provincial level) public health data exchange platform.

When a clinician diagnoses a notifiable chronic disease on his/her clinic system, a disease reporting card pops up automatically that presents data about that specific patient, which are extracted from the HIS or EHR. Clinicians only need to type in their diagnosis and treatment information to complete the whole report card, which takes only a few minutes. Health care doctors working in hospitals will then review the card to ensure the accuracy and logicality of the information. Besides, public health physicians who work in county/district CDC will double check the information afterward. If the content does not meet the standard requirements, the card will be returned to the health care doctors along with the feedback so as to be further edited. Once this has been done, the data will be transmitted to the regional health information platform immediately. Through a stepwise data exchange process, report cards will be transmitted to the Zhejiang Provincial Chronic Disease Surveillance Management System, and finally to the national system. Furthermore, these data will be exchanged with the Ningbo Digital Platform of Disease Control System and be shared across branch systems as shown in Figure 2. The process of data exchange and data sharing through a 3-level public health data exchange platform is presented in Figure 2. The data type collected on report card of notifiable NCDs is shown in Table 3. Data collected on regional health information platform include residents’ personal information, demographic characteristics, outpatient information, hospitalization information, results of examinations and laboratory tests, prescription lists, and immunization information. For municipal hospitals, the NCD cards reporting process is consistent with the reporting process of county hospital/health clinics/community service centers depicted in Figure 2.

Importantly, the purpose of building NCDCMS is not only to monitor the incidence of NCD but also to achieve better health management of patients with NCDs. Data exchange among platforms is not unidirectional, but rather a bidirectional data exchange process. When the NCD data are transmitted to the upper-level platform, cause of death and other branch surveillance system data (eg, infectious diseases, immunization records) of that particular patient will eventually be integrated into the EHR and transmitted back to the county/district regional information platform in order to achieve a two-way data exchange based on the principle of 1 person 1 file. Even if the NCD card is reported outside the place of residence, as long as it is reported within the Zhejiang Province, it will be returned to the place of residence following a bidirectional data exchange process.

Based on the stepwise 3-level public health data exchange platform, routine monitoring of 4 types of notifiable NCDs can be achieved on NCDCMS. Tables and charts can be automatically generated from surveillance data to track changes as needed.

**Table 2:** Four types of notifiable NCDs and their respective ICD-10 code(s).

Disease	ICD-10 code
Diabetes mellitus	E10-E14, O24
Ischemic heart disease and cardiac arrest	I20-I25, I46
Cerebrovascular disease	I60-I61, I63-I64
Malignant neoplasms	C00-C96
Benign neoplasm of the central nervous system	D32-D33, D42-D43

**Table 3:** Data types collected on report card of notifiable NCDs.

Diabetes mellitus	Ischemic heart disease and cardiac arrest	Cerebrovascular disease	Malignant neoplasms and benign neoplasm of the central nervous system
Electronic health record number	Electronic health record number	Electronic health record number	Electronic health record number
Patient name	Patient name	Patient name	Patient name
Gender	Gender	Gender	Gender
Nationality	Nationality	Nationality	Nationality
Education level	Education level	Education level	Education level
Identity card number	Identity card number	Identity card number	Identity card number
Date of birth	Date of birth	Date of birth	Date of birth
Occupation	Occupation	Occupation	Occupation
Contact number	Contact number	Contact number	Contact number
Marital status	Marital status	Marital status	Marital status
Age	Age	Age	Age
Registered address	Registered address	Registered address	Registered address
Current address	Current address	Current address	Current address
Outpatient number	Outpatient number	Outpatient number	Outpatient number
Hospitalization number	Hospitalization number	Hospitalization number	Hospitalization number
Report institution	Report institution	Report institution	Report institution
Report doctor name	Report doctor name	Report doctor name	Report doctor name
Date of report	Date of report	Date of report	Date of report
ICD-10 code of disease	ICD-10 code of disease	ICD-10 code of disease	ICD-10 code of disease
Disease type	Disease type	Disease type	Disease type
Date of diagnosis	Date of diagnosis	Date of diagnosis	Date of diagnosis
—^a^	Date of onset	Date of onset	—^a^
Symptoms	Symptoms	Symptoms	Symptoms
Examinations and tests	Examinations and tests	Examinations and tests	Examinations and tests
—^a^	—^a^	—^a^	ICD-O-3
—^a^	—^a^	—^a^	Pathologic type
—^a^	—^a^	—^a^	TMN stage
Complications	—^a^	—^a^	—^a^
Risk factors	—^a^	—^a^	—^a^
Family history	—^a^	—^a^	—^a^
—^a^	Medical history	Medical history	—^a^
Underlying cause of death	Underlying cause of death	Underlying cause of death	Underlying cause of death
Date of death	Date of death	Date of death	Date of death
ICD-10 code of death	ICD-10 code of death	ICD-10 code of death	ICD-10 code of death

^a^Not applicable.

### Evaluations of System Performance

#### Simplicity

Previously, during the data reporting process, clinicians first filled in NCD cards (either paper card or electronic card in HIS), and then health care doctors in hospitals manually entered the data into the Zhejiang Provincial Chronic Disease Surveillance Management System. Clinicians using electronic cards reported each patient information variable one by one (ie, on separate fields) into the HIS. It is well-known that manual data entry consumes a huge amount of human resources. Therefore, the NCDCMS was developed to offset the inefficiency of manual entries. Now, in the current system, when a clinician diagnoses a notifiable chronic disease on his/her clinic system, an NCD reporting card pops up automatically that presents much of the data about that specific patient, which are extracted from the HIS or EHR. Clinicians only need to type in their diagnosis and treatment information to complete the whole report card, which takes only a few minutes. Public health doctors in hospitals do not need to manually enter the reporting data in the surveillance system; instead, they only need to audit the report card and click the upload button, which transmits the data to the regional health information platform.

Meanwhile, because the NCDCMS realized the data collection process through a stepwise 3-level public health data exchange platform, which combined various independent platforms into one interconnected model, once a case has been reported, the data can be used in multiple ways and serve a variety of purposes. The data need to be reported by the clinicians only once and after the process is completed, the reported data can be transmitted directly to each platform in real time. This allows to eliminate duplicate entries.

#### Flexibility

To connect HIS with the data exchange platform, all medical institutions were required to adjust their public health module in HIS in accordance with the uniform data standards and the interface specifications; importantly, this adjustment is neither time consuming nor costly. In addition, the establishment of NCDCMS has a solid preliminary informatization foundation: (1) Ningbo is a city with a high level of informatization. (2) The 3-level direct reporting of notifiable infectious diseases by the 3-level public health data exchange platform was successfully achieved in 2016. (3) The key component of the system was structured by one company, which also increased the feasibility of the system to adapt to various requirements afterward. Because the adjustment of the public health module in HIS is neither time-consuming nor costly, with the solid informatization foundation of the whole city, the flexibility of the system can be guaranteed.

#### Data Quality

The quality of data collected by NCDCMS can be guaranteed in the following 4 aspects: (1) Each level of platform has a built-in logic check system and each type of NCD report card has several required items to be filled in. If the required items are not filled in completely or the contents of the report data do not meet the requirements of logical check, the report card information cannot be uploaded to the upper-level platform. (2) As mentioned above, a multilevel audit for all data reported will allow the data entered to be reviewed by health care doctors and public health physicians, which further ensures the accuracy of the collected data. (3) As the data platforms are directly related to each other, patient’s EHR file can be accessed during the case review process. In this way, data errors made by clinicians can be detected on time and accuracy of the data can be improved. (4) We perform data cleaning regularly and issue data analysis reports on a monthly, quarterly, and yearly basis to ensure that data quality meets the requirements. In addition, to ensure the authenticity of the reported data and have a continuous understanding of patients’ health status, a first visit within 1 month of data entry and a yearly follow-up of each NCD patient are conducted.

#### Acceptability

In the initial stages of the system construction, we selected 2 counties and 1 municipal-level hospital for model pilot tests. These early trials provided us with a great deal of experience in system construction. One year after the completion of the pilot tests, the system was gradually promoted and expanded to the whole city. This progressive approach of system construction also increased the acceptability of NCDCMS.

The construction of NCDCMS was government managed. The Health Commission of Ningbo explicitly demands every medical institution in Ningbo to complete the public health module adjustment to achieve the 3-level direct reporting of notifiable NCD diseases. Meanwhile, the completement of adjustment was considered as an indicator in the annual performance assessment of medical institutions. Therefore, China’s national conditions determine that this model can be widely accepted among medical institutions of Ningbo. At the time of this writing, among the 205 medical institutions that are required to report notifiable chronic diseases in Ningbo, 201 have completed the module adjustment, with the completion rate of 98.1% (201/205).

#### Representativeness

NCDCMS is totally based on the case reporting of medical institutions and has a facility coverage rate of 98.1% (201/205), meaning that as long as patients with NCDs visit medical institutions, either in hospital or in community, they will be reported by clinicians. Therefore, the NCD cases we collected via NCDCMS can reflect the actual situation of the whole population in Ningbo over time. In addition, based on the data collected by the system, the occurrence of 4 types of notifiable NCDs over time and their distribution in the population by place and person could be accurately described. Because of the continuous monitoring, the incidence trend of NCDs could be displayed and compared.

In addition to the NCDs data collected from NCDCMS, with the patient’s identity card number as a unique data matching criterion, more information can be drawn from other sources such as HIS and HER, which allows to build a more comprehensive database at the back end. Technically, from this comprehensive database, each patient’s data including his/her personal information, demographic characteristics, hospitalization information, results of examinations and tests, prescriptions, and survival situations could be obtained. Furthermore, branch surveillance systems are linked by the Ningbo Digital Platform of Disease Control and Prevention, and hence the data from these branch systems can also be shared and used for multiple purposes. From this perspective, using the data collected from Ningbo NCDCMS, the priorities of public health issues can be identified and the burden of disease can be measured in an accurate and convenient approach.

#### Timeliness

In the past, if a clinician reported an NCD card, irrespective of whether using a paper card or an electronic card, health care doctors working in hospitals had to manually enter report data variable one by one (ie, separately on each field) into the provincial chronic disease surveillance management system within 7 days. Although these cards are not immediately audited by county/district CDC staff, according to the registration and validation procedure established by provincial CDC, the staff are required to audit these cards within 7 days after data entry. Thus, this process, from initial data reporting on cards to the final completion of review, usually took several days, with a maximum duration of up to 14 days. However, because of escalating burden of NCDs in China, this procedure consumed a great deal of human resources, time, and costs.

Following the implementation of the new model, health care doctors’ working hours have been greatly reduced because of the removal of duplicate entries. Thus, more of their working time could be devoted to controlling the overall quality of the report card and training and improving the clinicians’ reporting skills. Thanks to the automatic popping up of the report card and information extraction approach, clinicians could fill in the report card when patients visit their clinic, or during the patient’s hospitalization, allowing simpler and faster completion. In addition, with the great improvement in the overall effectiveness of the new model, staff members from district/county CDC will review report cards on a daily basis. This new process greatly reduces the total time from reporting to reviewing of NCD cards, from the previous maximum of 14 days to the current minimum of 1 day. Compared with the past, the average card entry time is reduced from 10 minutes to 1 minute. Besides, the time taken for clinicians to enter relevant data at first visit and follow-up is reduced from 15 minutes to less than 1 minute, respectively. Excluding the time taken for manual auditing and reviewing, data are exchanged in real time between different platforms.

### Data Utilization

Using the data collected from NCDCMS and EHR, one study was conducted to describe the epidemiological characteristics of patients with diabetes mellitus (DM) diagnosed with oculopathy in one district in Ningbo. The diagnosed DM cases generated from NCDCMS were matched with the oculopathy cases generated from the regional health information platform by ID number and EHR number. The results showed that 1819 patients with DM were diagnosed with oculopathy in 2015. Among them, 195 patients with DM were newly diagnosed. The oculopathy comorbidity rate of the patients with DM and those newly diagnosed with DM in 2015 were 12.78% and 19.25%, respectively. The time intervals of patients with type 1 DM and type 2 DM from being diagnosed with diabetes to comorbidity of oculopathy was 4.00 and 3.00 years, respectively [18]. By linking diabetes cases and cancer cases generated from NCDCMS, one study implied that the patients with newly diagnosed type 2 DM, who were taller and had normal BMI, had a higher risk of cancer. Compared with their overweight (standardized incidence ratio 0.62) or obese (standardized incidence ratio 0.35) counterparts, the all-cause standardized incidence ratios of the normal BMI group was 1.13. Patients with normal BMI had a high risk of liver, pancreatic, esophagus, and ovary cancers [19].

Instead of field surveys, which consumed a large amount of human resource for data collection and analysis, the ecological studies and pilot epidemiological studies can be conducted directly through this 3-level public health data exchange platform. Moreover, we have started collecting NCD surveillance data since 2009 and each NCD case is required to be followed up once per year. These data will therefore contribute to carrying out different kinds of population-based cohort studies. Different variables needed for building cohorts can be obtained from multiple sources as mentioned earlier. Meanwhile, the information on all-cause mortality is also being collected, meaning that we can obtain data on the reliable survival situation of each patient with NCD. In one district of Ningbo, a cohort based on regional health information platform has been built to predict the risk factors of cardiovascular diseases [20]. Thus, big data tools can be applied for analyzing different data collected from the 3-level exchange platforms, with the results likely to be useful for decision making on various policies and strategies related to NCDs prevention and treatments.

## Discussion

To the best of our knowledge, NCDCMS is the first platform in China that achieved the 3-level direct reporting model based on regional health information platform. NCDCMS currently covers 201 medical institutions throughout the city, including municipal hospitals, county hospital, health clinics, and community service centers. The evaluations of the system performance showed that NCDCMS has high levels of simplicity, data quality, acceptability, representativeness, and timeliness. This 3-level direct reporting model helps relieve the burden of case reporting, as it allows real-time reporting, eliminates unnecessary redundancies, reduces the amount of underreporting, and provides a better database of chronic diseases.

The Ningbo Model is an innovative practice that aimed to develop a reliable population-based surveillance system via a real-time bidirectional 3-level data exchange process. It completely reshaped the traditional process of NCD surveillance data reporting and had three unique advantages: (1) It reduces the work burden of different stakeholders by data sharing and exchange. Previously, NCD reporting was completely dependent on manual data entry, which cannot guarantee the accuracy and was time-consuming. Now, clinicians could complete the case reporting during patient’s visit; thus, both the patient’s visit time and the work burden of clinicians are reduced due to the reduction of duplicated entries and repeated inquires. Besides, the workload of health care doctors who were responsible for checking the report card submitted by clinicians is reduced. (2) The amount of underreporting was greatly reduced through the automatic popping up of the report card. Some hospitals also link NCD reports with the discharge summary submission procedure. If the NCD cases are not reported as required, clinicians cannot eventually transfer the medical records of discharged patients to the medical record room. (3) The system contains better databases that allow data sharing among different platforms. A fairly complete personal health file can be obtained at the back end by linking different sources mentioned above. On this basis, the follow-up data, mortality data, and EHR data can be built into different kinds of NCD cohorts and the risk prediction models can be established. Different types of risk factors can be addressed and evidence-based interventions can also be performed. These research results can help policy makers to make decisions in an evidence-based way.

In the 2 years of NCDCMS construction, one difficulty was how to convince all medical institutions to use the uniform data standards and interface specifications. Because different medical institutions used different HISs developed by various companies, coordination among multiple stakeholders was initially expected to be difficult. In this aspect, the Health Commission of Ningbo and Ningbo CDC played important roles in facilitating the construction of the whole model. However, in order to obtain more accurate and reliable databases, a great amount of effort was still needed. Although we had the assistance of computer technology that helped avoid most logic mistakes in case reporting, a standardized training for case reporting was still important, especially for the clinicians who just started to use the system. The rapid development of health informatization also increased the need to establish better requirements for data security. Although the issue of underreporting has been greatly decreased, this situation could be reduced further with the progress of system development.

Considering the high level of health informatization in Ningbo, it seems that this model might be difficult to replicate in the provinces and cities with low level of health informatization. However, based on China’s current national conditions, there is an irresistible trend for improving informatization level in health industries nationwide, thus the feasibility of promoting and implementing this model nationwide is still achievable. After the completion of NCDCMS in 2018, the average number of visitors from the Health Commission and the CDCs located at different provinces and cities throughout the country was about 100 per year (as per data from Ningbo CDC), which also proved the enthusiasm for building health information platforms nationwide. At the time of writing, NCDCMS has been promoted and implemented in 5 cities in Zhejiang Province.

Driven by the development of health informatization in China, the Ningbo Model created a win–win situation for all stakeholders. Moreover, it actually enabled the possibility of performing population-based research with few human and material resources and helped achieve better data accessibility and utilization. The model has high replicability and generalizability in terms of standard establishment, workload reduction, elimination of underreporting, and improvement of data sharing and utilization. In China, it will guide the construction and establishment of systems for NCDs surveillance and management system in the future.

## Abbreviations

CDC: Centers for Disease Control and Prevention
COPD: chronic obstructive pulmonary disease
EHR: electronic health records
HIS: hospital information system
NCDCMS: the integrated noncommunicable disease collaborative management system
NCDs: noncommunicable diseases
